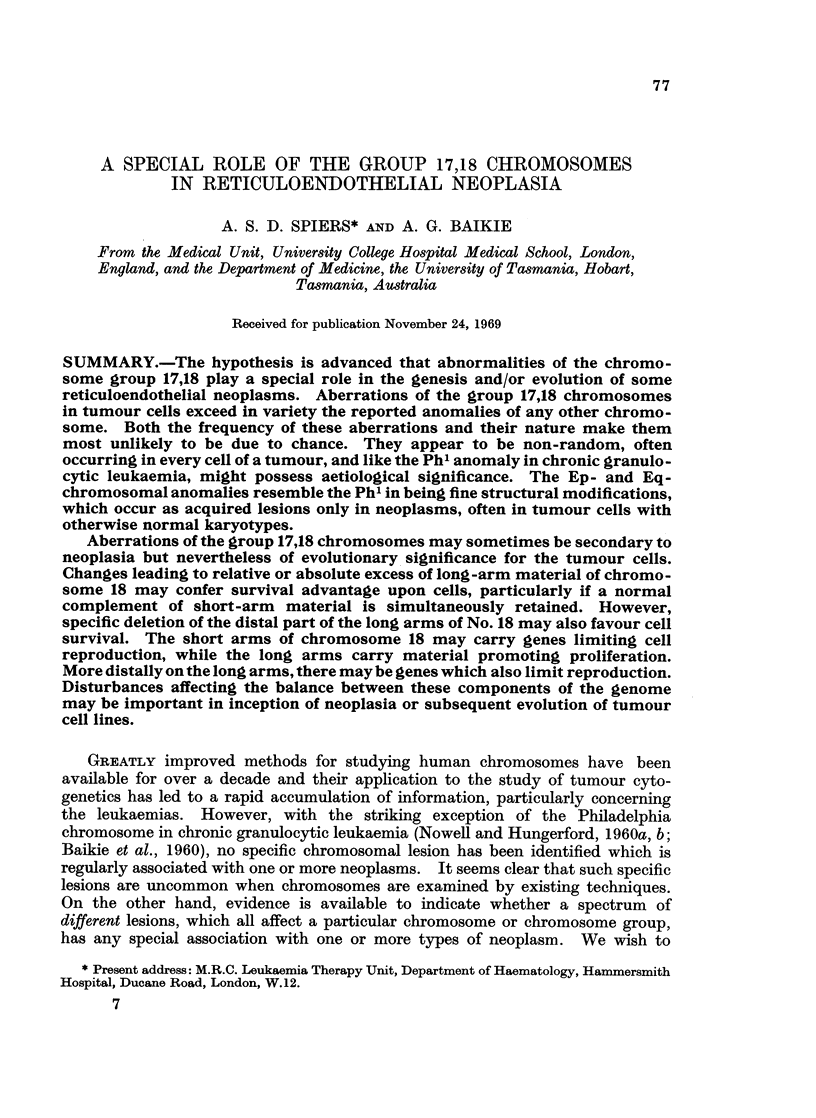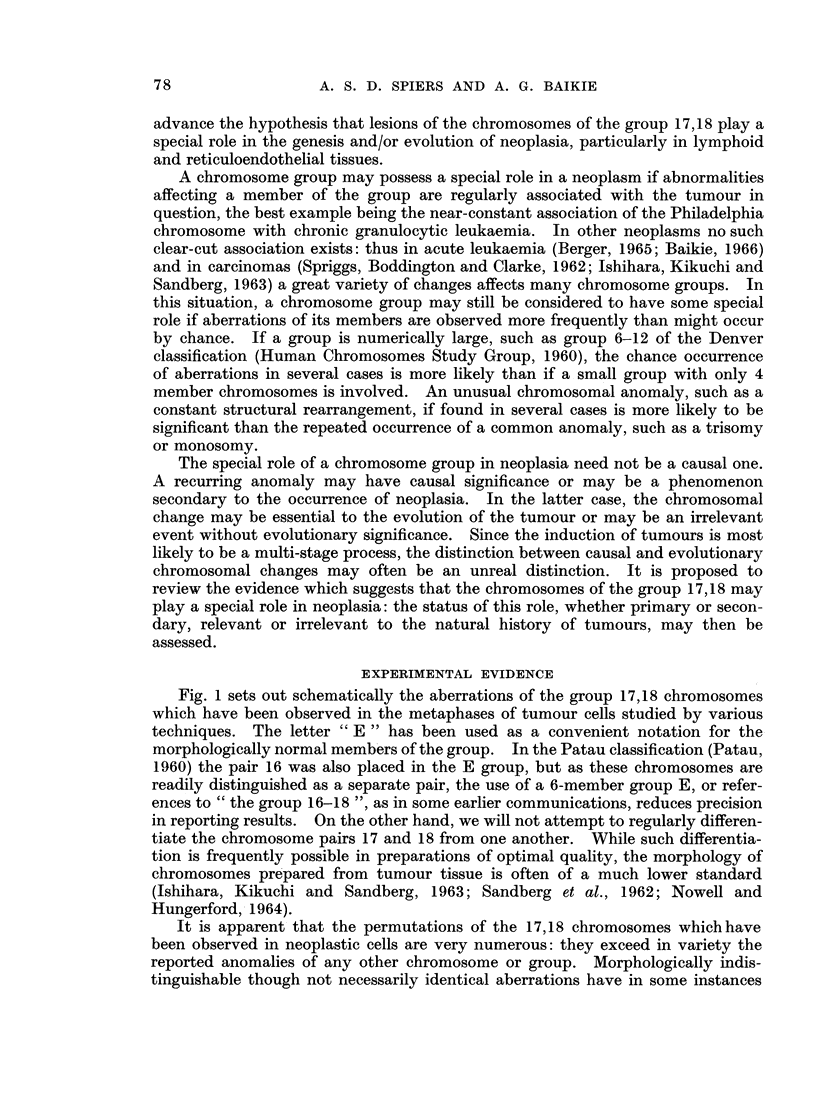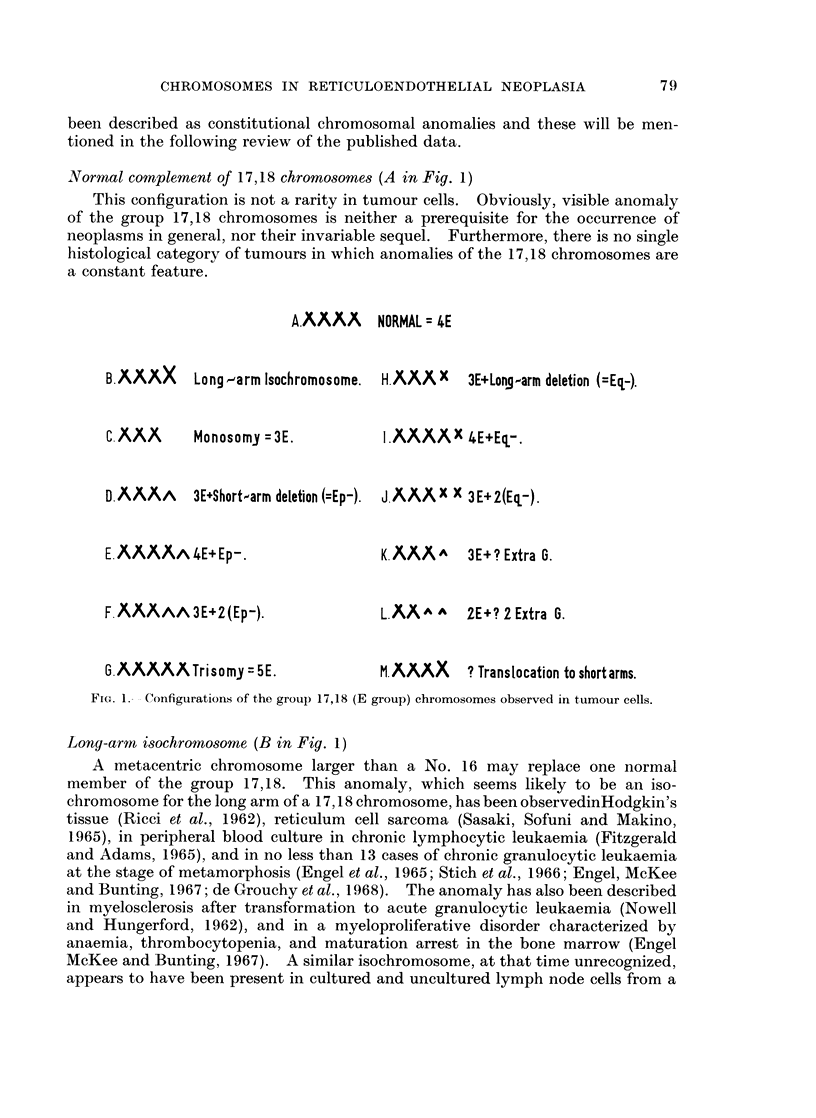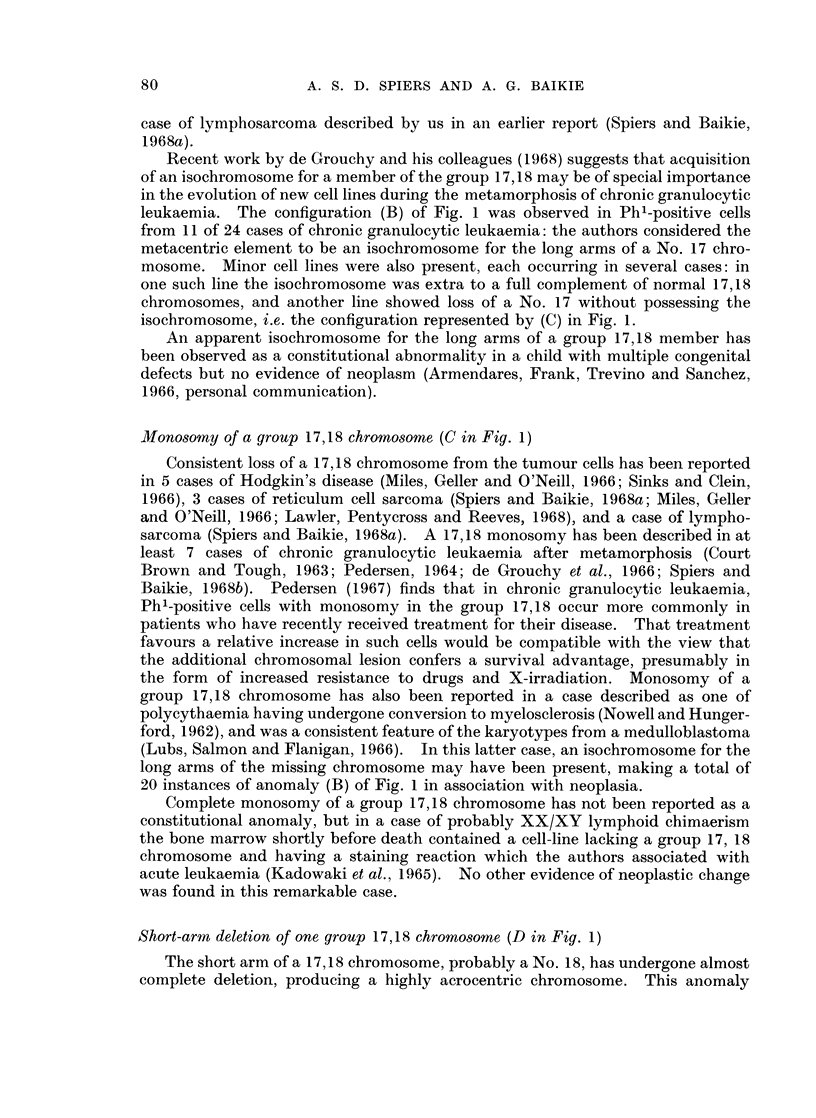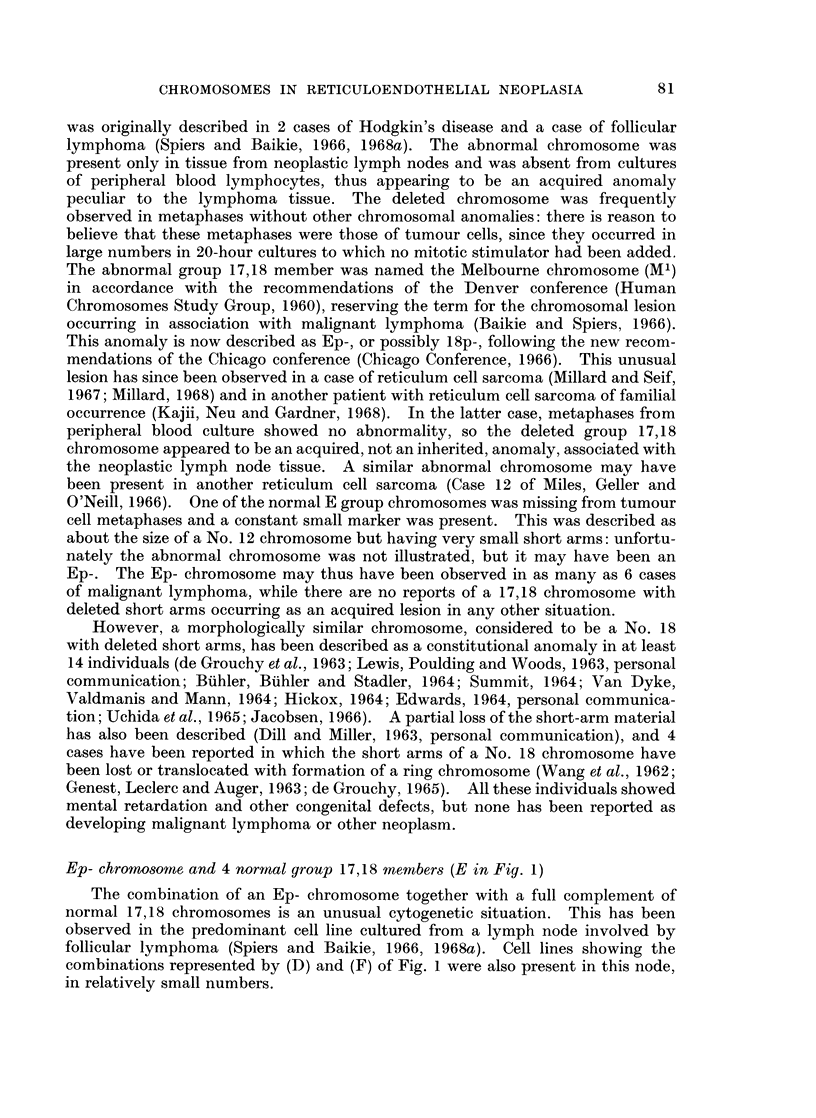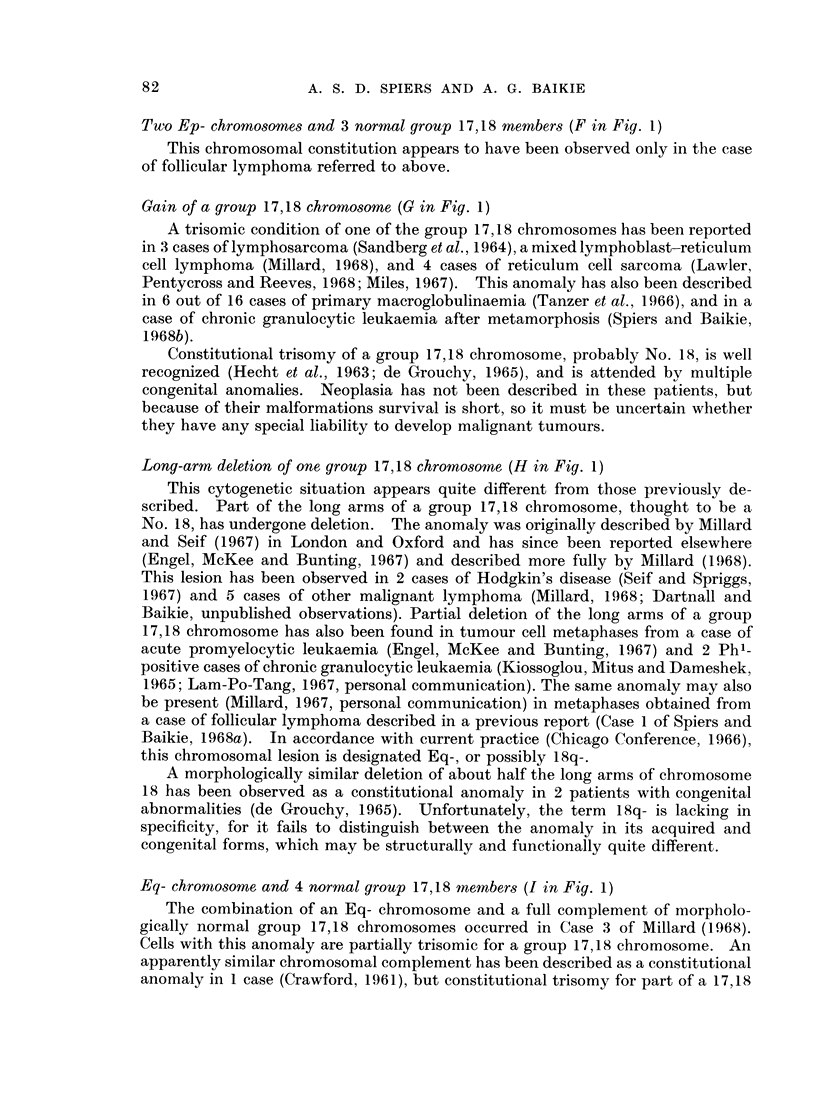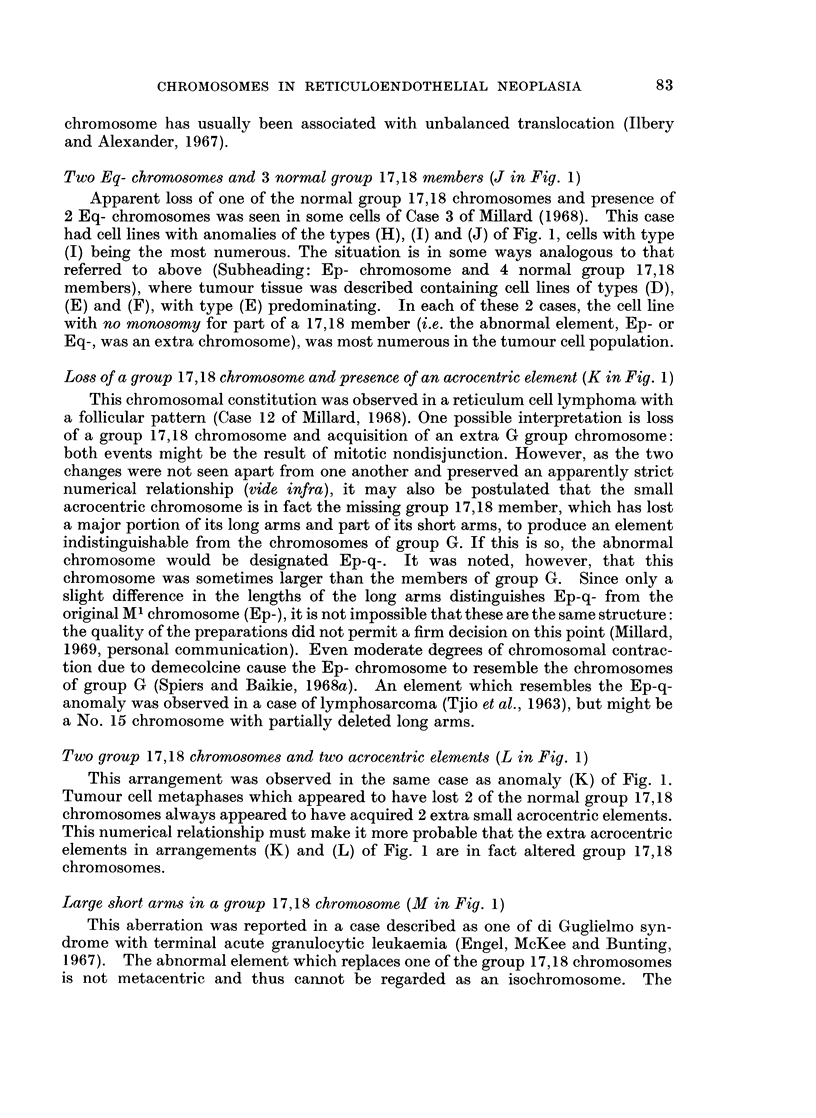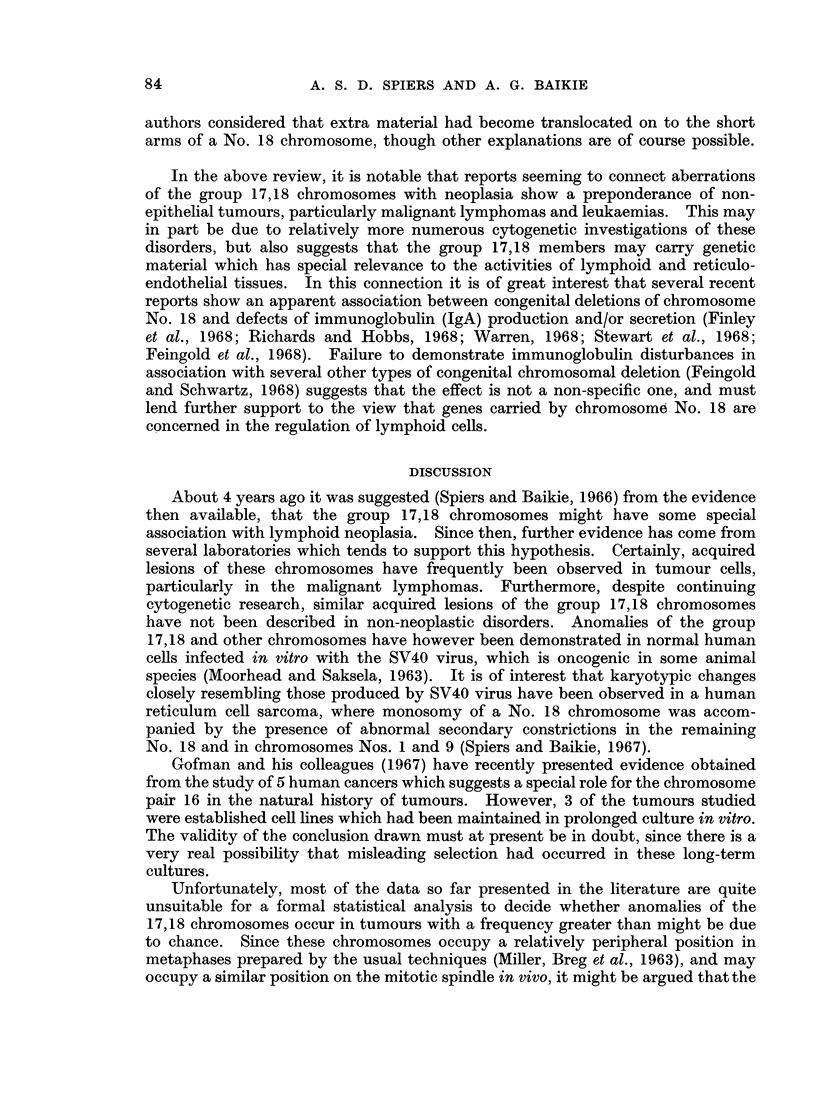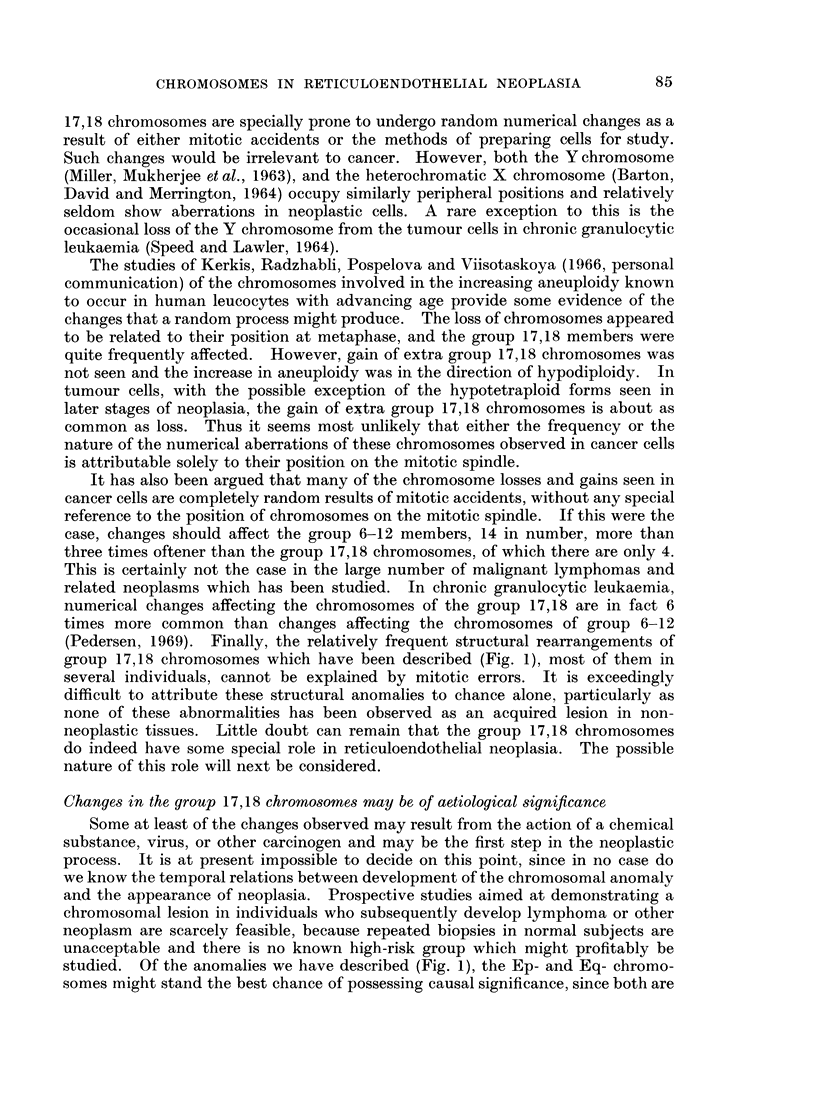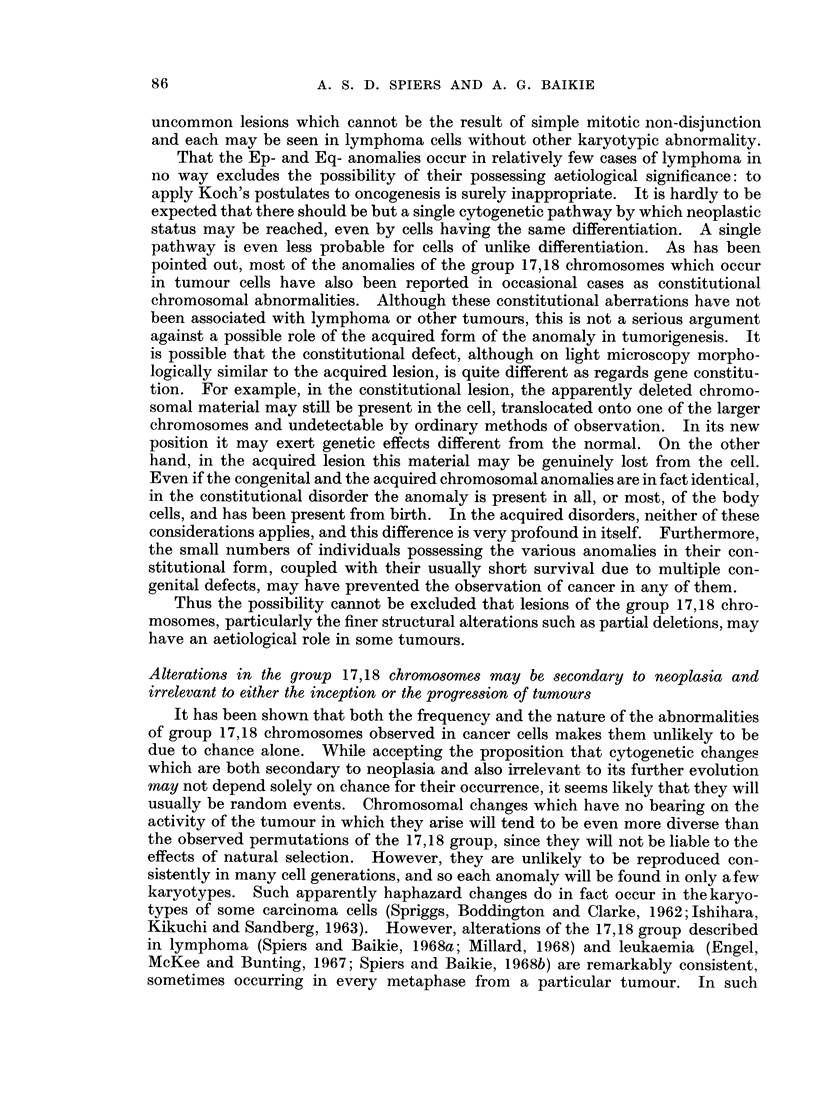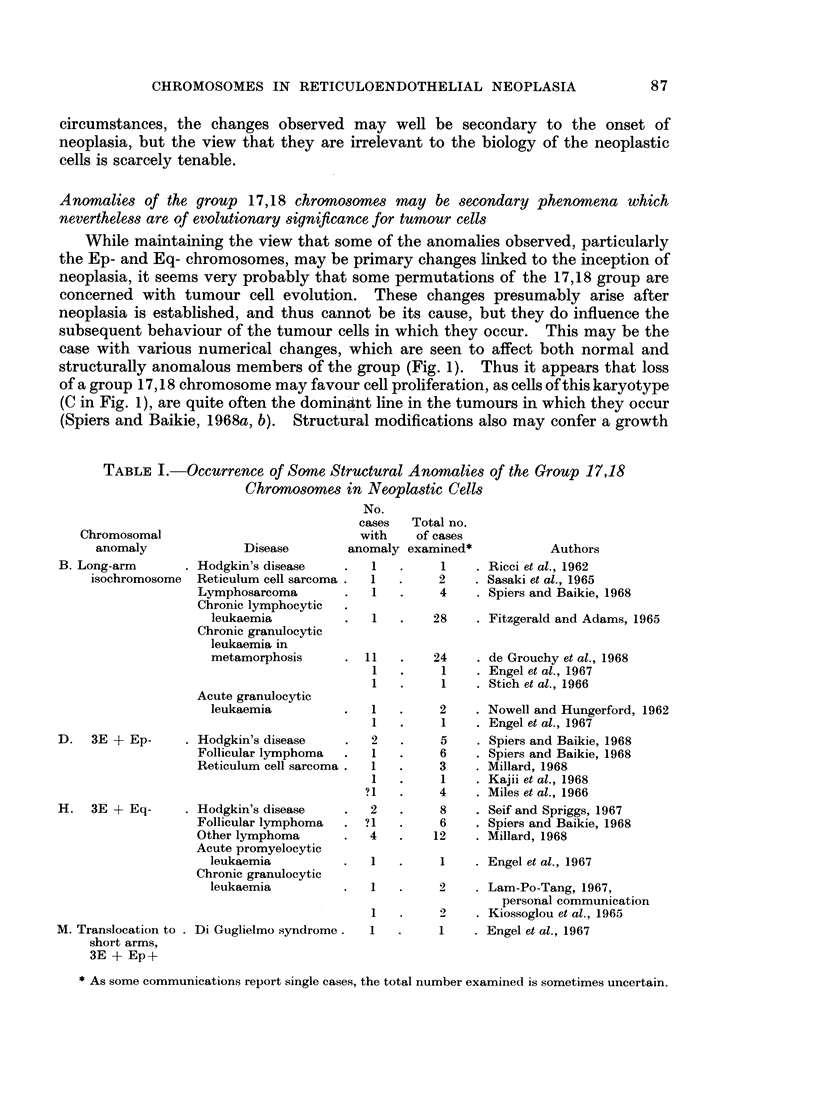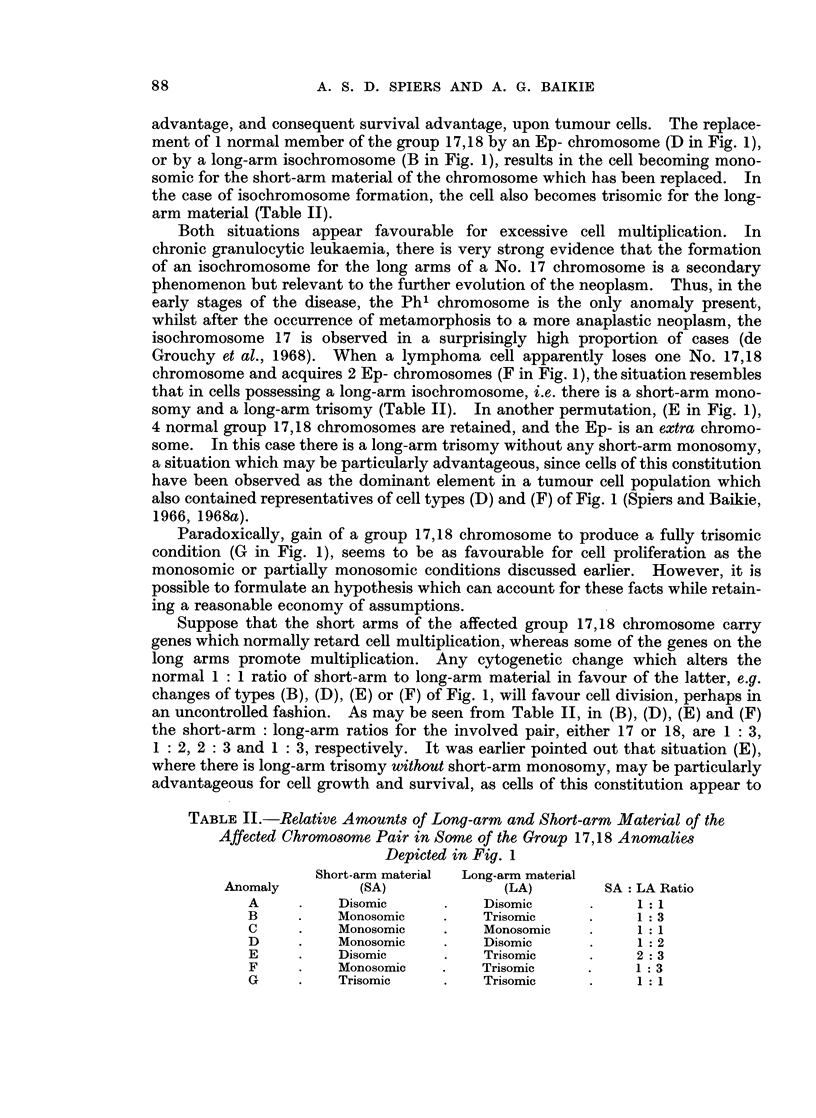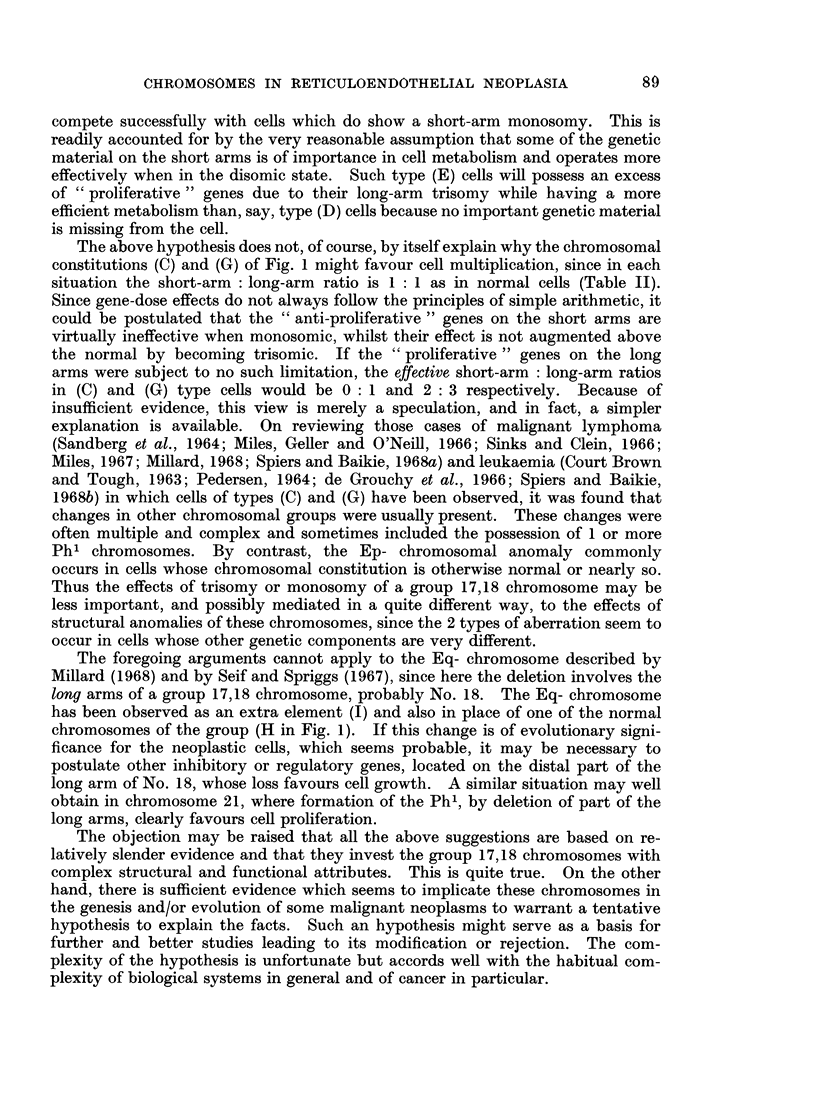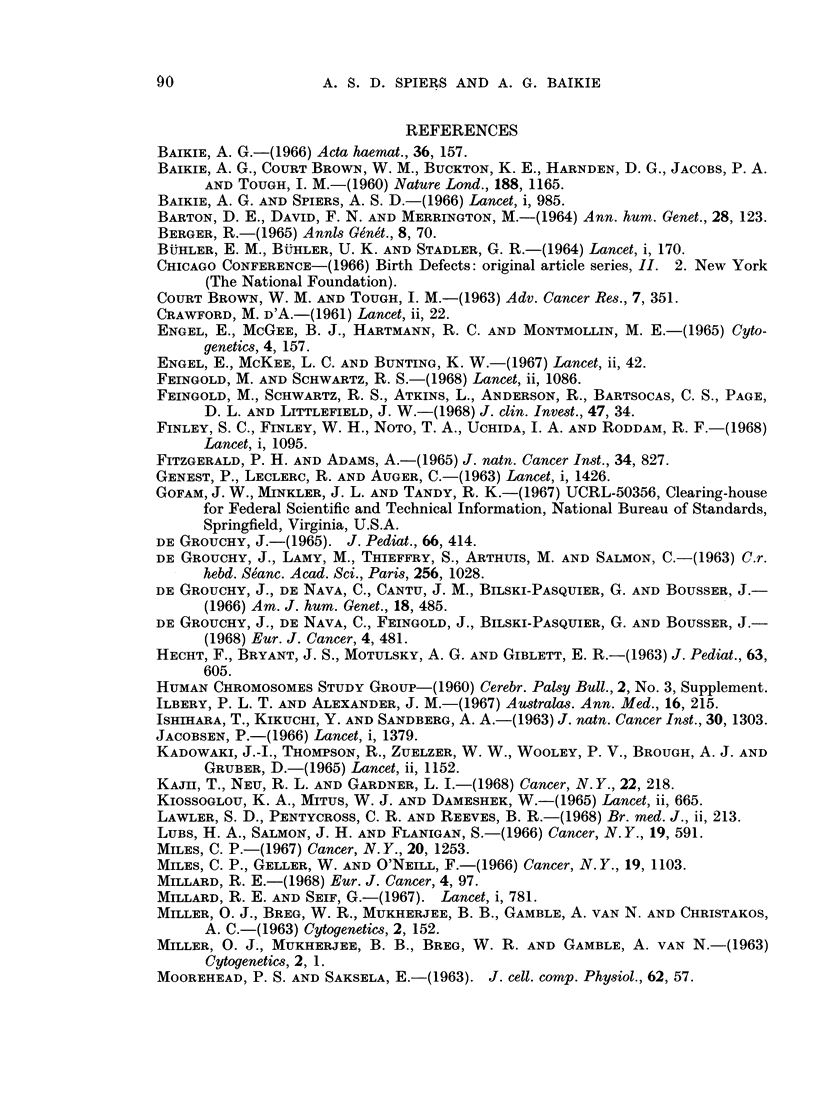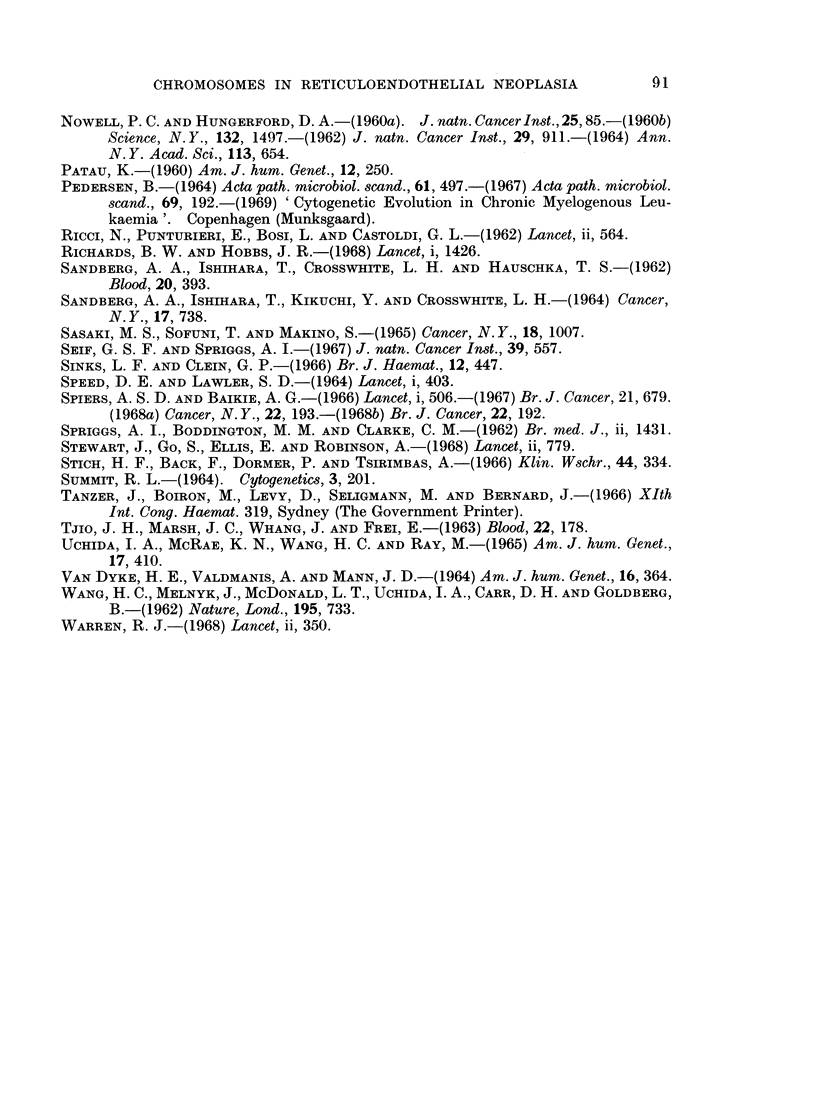# A Special Role of the Group 17,18 Chromosomes in Reticuloendothelial Neoplasia

**DOI:** 10.1038/bjc.1970.11

**Published:** 1970-03

**Authors:** A. S. D. Spiers, A. G. Baikie

## Abstract

The hypothesis is advanced that abnormalities of the chromosome group 17,18 play a special role in the genesis and/or evolution of some reticuloendothelial neoplasms. Aberrations of the group 17,18 chromosomes in tumour cells exceed in variety the reported anomaliesof any other chromosome. Both the frequency of these aberrations and their nature make them most unlikely to be due to chance. They appear to be non-random, often occurring in every cell of a tumour, and like the Ph^1^ anomaly in chronic granulocytic leukaemia, mightpossess aetiological significance. The Ep- and Eqchromosomal anomalies resemble the Ph^1^ in being fine structural modifications, which occur as acquired lesions only in neoplasms, often in tumour cells with otherwise normal karyotypes.

Aberration of the group 17,18 chromosomes may sometimes be secondary to neoplasia but nevertheless of evolutionary significance for the tumour cells. Changes leading to relative or absolute excess of long-arm material of chromosome 18 may confer survival advantage upon cells, particularly if a normal complement of short-arm material is simultaneously retained. However, specific deletion of the distal part of the long arms of No. 18 may also favour cell survival. The short arms of chromosome 18 may carry genes limiting cell reproduction, while the long arms carry material promoting proliferation. More distally on the long arms, there may be genes which also limit reproduction. Disturbances affecting the balance between these components of the genome may be important in inception of neoplasia or subsequent evolution of tumour cell lines.


### A SPECIAL ROLE OF THE GROUP 17,18 CHROMOSOMES IN RETICULOENDOTHELIAL NEOPLASIA

A. S. D. SPIERS* AND A. G. BAIKIE

*From the Medical Unit, University College Hospital Medical School, London, England, and the Department of Medicine, the University of Tasmania, Hobart, Tasmania, Australia*

Received for publication November 24, 1969

**SUMMARY.—The hypothesis is advanced that abnormalities of the chromosome group 17,18 play a special role in the genesis and/or evolution of some reticuloendothelial neoplasms. Aberrations of the group 17,18 chromosomes in tumour cells exceed in variety the reported anomalies of any other chromosome. Both the frequency of these aberrations and their nature make them most unlikely to be due to chance. They appear to be non-random, often occurring in every cell of a tumour, and like the $Ph^1$ anomaly in chronic granulocytic leukaemia, might possess aetiological significance. The Ep- and Eq- chromosomal anomalies resemble the $Ph^1$ in being fine structural modifications, which occur as acquired lesions only in neoplasms, often in tumour cells with otherwise normal karyotypes.**

**Aberrations of the group 17,18 chromosomes may sometimes be secondary to neoplasia but nevertheless of evolutionary significance for the tumour cells. Changes leading to relative or absolute excess of long-arm material of chromosome 18 may confer survival advantage upon cells, particularly if a normal complement of short-arm material is simultaneously retained. However, specific deletion of the distal part of the long arms of No. 18 may also favour cell survival. The short arms of chromosome 18 may carry genes limiting cell reproduction, while the long arms carry material promoting proliferation. More distally on the long arms, there may be genes which also limit reproduction. Disturbances affecting the balance between these components of the genome may be important in inception of neoplasia or subsequent evolution of tumour cell lines.**

GREATLY improved methods for studying human chromosomes have been available for over a decade and their application to the study of tumour cytogenetics has led to a rapid accumulation of information, particularly concerning the leukaemias. However, with the striking exception of the Philadelphia chromosome in chronic granulocytic leukaemia (Nowell and Hungerford, 1960*a*, *b*; Baikie *et al.*, 1960), no specific chromosomal lesion has been identified which is regularly associated with one or more neoplasms. It seems clear that such specific lesions are uncommon when chromosomes are examined by existing techniques. On the other hand, evidence is available to indicate whether a spectrum of *different* lesions, which all affect a particular chromosome or chromosome group, has any special association with one or more types of neoplasm. We wish to

* Present address: M.R.C. Leukaemia Therapy Unit, Department of Haematology, Hammersmith Hospital, Ducane Road, London, W.12.

advance the hypothesis that lesions of the chromosomes of the group 17,18 play a special role in the genesis and/or evolution of neoplasia, particularly in lymphoid and reticuloendothelial tissues.

A chromosome group may possess a special role in a neoplasm if abnormalities affecting a member of the group are regularly associated with the tumour in question, the best example being the near-constant association of the Philadelphia chromosome with chronic granulocytic leukaemia. In other neoplasms no such clear-cut association exists: thus in acute leukaemia (Berger, 1965; Baikie, 1966) and in carcinomas (Spriggs, Boddington and Clarke, 1962; Ishihara, Kikuchi and Sandberg, 1963) a great variety of changes affects many chromosome groups. In this situation, a chromosome group may still be considered to have some special role if aberrations of its members are observed more frequently than might occur by chance. If a group is numerically large, such as group 6–12 of the Denver classification (Human Chromosomes Study Group, 1960), the chance occurrence of aberrations in several cases is more likely than if a small group with only 4 member chromosomes is involved. An unusual chromosomal anomaly, such as a constant structural rearrangement, if found in several cases is more likely to be significant than the repeated occurrence of a common anomaly, such as a trisomy or monosomy.

The special role of a chromosome group in neoplasia need not be a causal one. A recurring anomaly may have causal significance or may be a phenomenon secondary to the occurrence of neoplasia. In the latter case, the chromosomal change may be essential to the evolution of the tumour or may be an irrelevant event without evolutionary significance. Since the induction of tumours is most likely to be a multi-stage process, the distinction between causal and evolutionary chromosomal changes may often be an unreal distinction. It is proposed to review the evidence which suggests that the chromosomes of the group 17,18 may play a special role in neoplasia: the status of this role, whether primary or secondary, relevant or irrelevant to the natural history of tumours, may then be assessed.

### EXPERIMENTAL EVIDENCE

Fig. 1 sets out schematically the aberrations of the group 17,18 chromosomes which have been observed in the metaphases of tumour cells studied by various techniques. The letter " E " has been used as a convenient notation for the morphologically normal members of the group. In the Patau classification (Patau, 1960) the pair 16 was also placed in the E group, but as these chromosomes are readily distinguished as a separate pair, the use of a 6-member group E, or references to " the group 16–18 ", as in some earlier communications, reduces precision in reporting results. On the other hand, we will not attempt to regularly differentiate the chromosome pairs 17 and 18 from one another. While such differentiation is frequently possible in preparations of optimal quality, the morphology of chromosomes prepared from tumour tissue is often of a much lower standard (Ishihara, Kikuchi and Sandberg, 1963; Sandberg *et al.*, 1962; Nowell and Hungerford, 1964).

It is apparent that the permutations of the 17,18 chromosomes which have been observed in neoplastic cells are very numerous: they exceed in variety the reported anomalies of any other chromosome or group. Morphologically indistinguishable though not necessarily identical aberrations have in some instances been described as constitutional chromosomal anomalies and these will be mentioned in the following review of the published data.

*Normal complement of* 17,18 *chromosomes* (*A in Fig.* 1)

This configuration is not a rarity in tumour cells. Obviously, visible anomaly of the group 17,18 chromosomes is neither a prerequisite for the occurrence of neoplasms in general, nor their invariable sequel. Furthermore, there is no single histological category of tumours in which anomalies of the 17,18 chromosomes are a constant feature.

FIG. 1.—Configurations of the group 17,18 (E group) chromosomes observed in tumour cells.

*Long-arm isochromosome* (*B in Fig.* 1)

A metacentric chromosome larger than a No. 16 may replace one normal member of the group 17,18. This anomaly, which seems likely to be an isochromosome for the long arm of a 17,18 chromosome, has been observedinHodgkin's tissue (Ricci *et al.*, 1962), reticulum cell sarcoma (Sasaki, Sofuni and Makino, 1965), in peripheral blood culture in chronic lymphocytic leukaemia (Fitzgerald and Adams, 1965), and in no less than 13 cases of chronic granulocytic leukaemia at the stage of metamorphosis (Engel *et al.*, 1965; Stich *et al.*, 1966; Engel, McKee and Bunting, 1967; de Grouchy *et al.*, 1968). The anomaly has also been described in myelosclerosis after transformation to acute granulocytic leukaemia (Nowell and Hungerford, 1962), and in a myeloproliferative disorder characterized by anaemia, thrombocytopenia, and maturation arrest in the bone marrow (Engel McKee and Bunting, 1967). A similar isochromosome, at that time unrecognized, appears to have been present in cultured and uncultured lymph node cells from a case of lymphosarcoma described by us in an earlier report (Spiers and Baikie, 1968*a*).

Recent work by de Grouchy and his colleagues (1968) suggests that acquisition of an isochromosome for a member of the group 17,18 may be of special importance in the evolution of new cell lines during the metamorphosis of chronic granulocytic leukaemia. The configuration (B) of Fig. 1 was observed in $Ph^1$-positive cells from 11 of 24 cases of chronic granulocytic leukaemia: the authors considered the metacentric element to be an isochromosome for the long arms of a No. 17 chromosome. Minor cell lines were also present, each occurring in several cases: in one such line the isochromosome was extra to a full complement of normal 17,18 chromosomes, and another line showed loss of a No. 17 without possessing the isochromosome, *i.e.* the configuration represented by (C) in Fig. 1.

An apparent isochromosome for the long arms of a group 17,18 member has been observed as a constitutional abnormality in a child with multiple congenital defects but no evidence of neoplasm (Armendares, Frank, Trevino and Sanchez, 1966, personal communication).

*Monosomy of a group* 17,18 *chromosome* (*C in Fig.* 1)

Consistent loss of a 17,18 chromosome from the tumour cells has been reported in 5 cases of Hodgkin's disease (Miles, Geller and O'Neill, 1966; Sinks and Clein, 1966), 3 cases of reticulum cell sarcoma (Spiers and Baikie, 1968*a*; Miles, Geller and O'Neill, 1966; Lawler, Pentycross and Reeves, 1968), and a case of lymphosarcoma (Spiers and Baikie, 1968*a*). A 17,18 monosomy has been described in at least 7 cases of chronic granulocytic leukaemia after metamorphosis (Court Brown and Tough, 1963; Pedersen, 1964; de Grouchy *et al.*, 1966; Spiers and Baikie, 1968*b*). Pedersen (1967) finds that in chronic granulocytic leukaemia, $Ph^1$-positive cells with monosomy in the group 17,18 occur more commonly in patients who have recently received treatment for their disease. That treatment favours a relative increase in such cells would be compatible with the view that the additional chromosomal lesion confers a survival advantage, presumably in the form of increased resistance to drugs and X-irradiation. Monosomy of a group 17,18 chromosome has also been reported in a case described as one of polycythaemia having undergone conversion to myelosclerosis (Nowell and Hungerford, 1962), and was a consistent feature of the karyotypes from a medulloblastoma (Lubs, Salmon and Flanigan, 1966). In this latter case, an isochromosome for the long arms of the missing chromosome may have been present, making a total of 20 instances of anomaly (B) of Fig. 1 in association with neoplasia.

Complete monosomy of a group 17,18 chromosome has not been reported as a constitutional anomaly, but in a case of probably XX/XY lymphoid chimaerism the bone marrow shortly before death contained a cell-line lacking a group 17, 18 chromosome and having a staining reaction which the authors associated with acute leukaemia (Kadowaki *et al.*, 1965). No other evidence of neoplastic change was found in this remarkable case.

*Short-arm deletion of one group* 17,18 *chromosome* (*D in Fig.* 1)

The short arm of a 17,18 chromosome, probably a No. 18, has undergone almost complete deletion, producing a highly acrocentric chromosome. This anomaly was originally described in 2 cases of Hodgkin's disease and a case of follicular lymphoma (Spiers and Baikie, 1966, 1968*a*). The abnormal chromosome was present only in tissue from neoplastic lymph nodes and was absent from cultures of peripheral blood lymphocytes, thus appearing to be an acquired anomaly peculiar to the lymphoma tissue. The deleted chromosome was frequently observed in metaphases without other chromosomal anomalies: there is reason to believe that these metaphases were those of tumour cells, since they occurred in large numbers in 20-hour cultures to which no mitotic stimulator had been added. The abnormal group 17,18 member was named the Melbourne chromosome ($M^1$) in accordance with the recommendations of the Denver conference (Human Chromosomes Study Group, 1960), reserving the term for the chromosomal lesion occurring in association with malignant lymphoma (Baikie and Spiers, 1966). This anomaly is now described as Ep-, or possibly 18p-, following the new recommendations of the Chicago conference (Chicago Conference, 1966). This unusual lesion has since been observed in a case of reticulum cell sarcoma (Millard and Seif, 1967; Millard, 1968) and in another patient with reticulum cell sarcoma of familial occurrence (Kajii, Neu and Gardner, 1968). In the latter case, metaphases from peripheral blood culture showed no abnormality, so the deleted group 17,18 chromosome appeared to be an acquired, not an inherited, anomaly, associated with the neoplastic lymph node tissue. A similar abnormal chromosome may have been present in another reticulum cell sarcoma (Case 12 of Miles, Geller and O'Neill, 1966). One of the normal E group chromosomes was missing from tumour cell metaphases and a constant small marker was present. This was described as about the size of a No. 12 chromosome but having very small short arms: unfortunately the abnormal chromosome was not illustrated, but it may have been an Ep-. The Ep- chromosome may thus have been observed in as many as 6 cases of malignant lymphoma, while there are no reports of a 17,18 chromosome with deleted short arms occurring as an acquired lesion in any other situation.

However, a morphologically similar chromosome, considered to be a No. 18 with deleted short arms, has been described as a constitutional anomaly in at least 14 individuals (de Grouchy *et al.*, 1963; Lewis, Poulding and Woods, 1963, personal communication; Bühler, Bühler and Stadler, 1964; Summit, 1964; Van Dyke, Valdmanis and Mann, 1964; Hickox, 1964; Edwards, 1964, personal communication; Uchida *et al.*, 1965; Jacobsen, 1966). A partial loss of the short-arm material has also been described (Dill and Miller, 1963, personal communication), and 4 cases have been reported in which the short arms of a No. 18 chromosome have been lost or translocated with formation of a ring chromosome (Wang *et al.*, 1962; Genest, Leclerc and Auger, 1963; de Grouchy, 1965). All these individuals showed mental retardation and other congenital defects, but none has been reported as developing malignant lymphoma or other neoplasm.

*Ep- chromosome and* 4 *normal group* 17,18 *members* (*E in Fig.* 1)

The combination of an Ep- chromosome together with a full complement of normal 17,18 chromosomes is an unusual cytogenetic situation. This has been observed in the predominant cell line cultured from a lymph node involved by follicular lymphoma (Spiers and Baikie, 1966, 1968*a*). Cell lines showing the combinations represented by (D) and (F) of Fig. 1 were also present in this node, in relatively small numbers.

*Two Ep- chromosomes and* 3 *normal group* 17,18 *members* (*F in Fig.* 1)

This chromosomal constitution appears to have been observed only in the case of follicular lymphoma referred to above.

*Gain of a group* 17,18 *chromosome* (*G in Fig.* 1)

A trisomic condition of one of the group 17,18 chromosomes has been reported in 3 cases of lymphosarcoma (Sandberg *et al.*, 1964), a mixed lymphoblast–reticulum cell lymphoma (Millard, 1968), and 4 cases of reticulum cell sarcoma (Lawler, Pentycross and Reeves, 1968; Miles, 1967). This anomaly has also been described in 6 out of 16 cases of primary macroglobulinaemia (Tanzer *et al.*, 1966), and in a case of chronic granulocytic leukaemia after metamorphosis (Spiers and Baikie, 1968*b*).

Constitutional trisomy of a group 17,18 chromosome, probably No. 18, is well recognized (Hecht *et al.*, 1963; de Grouchy, 1965), and is attended by multiple congenital anomalies. Neoplasia has not been described in these patients, but because of their malformations survival is short, so it must be uncertain whether they have any special liability to develop malignant tumours.

*Long-arm deletion of one group* 17,18 *chromosome* (*H in Fig.* 1)

This cytogenetic situation appears quite different from those previously described. Part of the long arms of a group 17,18 chromosome, thought to be a No. 18, has undergone deletion. The anomaly was originally described by Millard and Seif (1967) in London and Oxford and has since been reported elsewhere (Engel, McKee and Bunting, 1967) and described more fully by Millard (1968). This lesion has been observed in 2 cases of Hodgkin's disease (Seif and Spriggs, 1967) and 5 cases of other malignant lymphoma (Millard, 1968; Dartnall and Baikie, unpublished observations). Partial deletion of the long arms of a group 17,18 chromosome has also been found in tumour cell metaphases from a case of acute promyelocytic leukaemia (Engel, McKee and Bunting, 1967) and 2 $Ph^1$-positive cases of chronic granulocytic leukaemia (Kiossoglou, Mitus and Dameshek, 1965; Lam-Po-Tang, 1967, personal communication). The same anomaly may also be present (Millard, 1967, personal communication) in metaphases obtained from a case of follicular lymphoma described in a previous report (Case 1 of Spiers and Baikie, 1968*a*). In accordance with current practice (Chicago Conference, 1966), this chromosomal lesion is designated Eq-, or possibly 18q-.

A morphologically similar deletion of about half the long arms of chromosome 18 has been observed as a constitutional anomaly in 2 patients with congenital abnormalities (de Grouchy, 1965). Unfortunately, the term 18q- is lacking in specificity, for it fails to distinguish between the anomaly in its acquired and congenital forms, which may be structurally and functionally quite different.

*Eq- chromosome and* 4 *normal group* 17,18 *members* (*I in Fig.* 1)

The combination of an Eq- chromosome and a full complement of morphologically normal group 17,18 chromosomes occurred in Case 3 of Millard (1968). Cells with this anomaly are partially trisomic for a group 17,18 chromosome. An apparently similar chromosomal complement has been described as a constitutional anomaly in 1 case (Crawford, 1961), but constitutional trisomy for part of a 17,18

chromosome has usually been associated with unbalanced translocation (Ilbery and Alexander, 1967).

*Two Eq- chromosomes and* 3 *normal group* 17,18 *members* (*J in Fig.* 1)

Apparent loss of one of the normal group 17,18 chromosomes and presence of 2 Eq- chromosomes was seen in some cells of Case 3 of Millard (1968). This case had cell lines with anomalies of the types (H), (I) and (J) of Fig. 1, cells with type (I) being the most numerous. The situation is in some ways analogous to that referred to above (Subheading: Ep- chromosome and 4 normal group 17,18 members), where tumour tissue was described containing cell lines of types (D), (E) and (F), with type (E) predominating. In each of these 2 cases, the cell line with *no monosomy* for part of a 17,18 member (*i.e.* the abnormal element, Ep- or Eq-, was an extra chromosome), was most numerous in the tumour cell population.

*Loss of a group* 17,18 *chromosome and presence of an acrocentric element* (*K in Fig.* 1)

This chromosomal constitution was observed in a reticulum cell lymphoma with a follicular pattern (Case 12 of Millard, 1968). One possible interpretation is loss of a group 17,18 chromosome and acquisition of an extra G group chromosome: both events might be the result of mitotic nondisjunction. However, as the two changes were not seen apart from one another and preserved an apparently strict numerical relationship (*vide infra*), it may also be postulated that the small acrocentric chromosome is in fact the missing group 17,18 member, which has lost a major portion of its long arms and part of its short arms, to produce an element indistinguishable from the chromosomes of group G. If this is so, the abnormal chromosome would be designated Ep-q-. It was noted, however, that this chromosome was sometimes larger than the members of group G. Since only a slight difference in the lengths of the long arms distinguishes Ep-q- from the original $M^1$ chromosome (Ep-), it is not impossible that these are the same structure: the quality of the preparations did not permit a firm decision on this point (Millard, 1969, personal communication). Even moderate degrees of chromosomal contraction due to demecolcine cause the Ep- chromosome to resemble the chromosomes of group G (Spiers and Baikie, 1968*a*). An element which resembles the Ep-q- anomaly was observed in a case of lymphosarcoma (Tjio *et al.*, 1963), but might be a No. 15 chromosome with partially deleted long arms.

*Two group* 17,18 *chromosomes and two acrocentric elements* (*L in Fig.* 1)

This arrangement was observed in the same case as anomaly (K) of Fig. 1. Tumour cell metaphases which appeared to have lost 2 of the normal group 17,18 chromosomes always appeared to have acquired 2 extra small acrocentric elements. This numerical relationship must make it more probable that the extra acrocentric elements in arrangements (K) and (L) of Fig. 1 are in fact altered group 17,18 chromosomes.

*Large short arms in a group* 17,18 *chromosome* (*M in Fig.* 1)

This aberration was reported in a case described as one of di Guglielmo syndrome with terminal acute granulocytic leukaemia (Engel, McKee and Bunting, 1967). The abnormal element which replaces one of the group 17,18 chromosomes is not metacentric and thus cannot be regarded as an isochromosome. The authors considered that extra material had become translocated on to the short arms of a No. 18 chromosome, though other explanations are of course possible.

In the above review, it is notable that reports seeming to connect aberrations of the group 17,18 chromosomes with neoplasia show a preponderance of non-epithelial tumours, particularly malignant lymphomas and leukaemias. This may in part be due to relatively more numerous cytogenetic investigations of these disorders, but also suggests that the group 17,18 members may carry genetic material which has special relevance to the activities of lymphoid and reticulo-endothelial tissues. In this connection it is of great interest that several recent reports show an apparent association between congenital deletions of chromosome No. 18 and defects of immunoglobulin (IgA) production and/or secretion (Finley *et al.*, 1968; Richards and Hobbs, 1968; Warren, 1968; Stewart *et al.*, 1968; Feingold *et al.*, 1968). Failure to demonstrate immunoglobulin disturbances in association with several other types of congenital chromosomal deletion (Feingold and Schwartz, 1968) suggests that the effect is not a non-specific one, and must lend further support to the view that genes carried by chromosome No. 18 are concerned in the regulation of lymphoid cells.

### DISCUSSION

About 4 years ago it was suggested (Spiers and Baikie, 1966) from the evidence then available, that the group 17,18 chromosomes might have some special association with lymphoid neoplasia. Since then, further evidence has come from several laboratories which tends to support this hypothesis. Certainly, acquired lesions of these chromosomes have frequently been observed in tumour cells, particularly in the malignant lymphomas. Furthermore, despite continuing cytogenetic research, similar acquired lesions of the group 17,18 chromosomes have not been described in non-neoplastic disorders. Anomalies of the group 17,18 and other chromosomes have however been demonstrated in normal human cells infected *in vitro* with the SV40 virus, which is oncogenic in some animal species (Moorhead and Saksela, 1963). It is of interest that karyotypic changes closely resembling those produced by SV40 virus have been observed in a human reticulum cell sarcoma, where monosomy of a No. 18 chromosome was accompanied by the presence of abnormal secondary constrictions in the remaining No. 18 and in chromosomes Nos. 1 and 9 (Spiers and Baikie, 1967).

Gofman and his colleagues (1967) have recently presented evidence obtained from the study of 5 human cancers which suggests a special role for the chromosome pair 16 in the natural history of tumours. However, 3 of the tumours studied were established cell lines which had been maintained in prolonged culture *in vitro*. The validity of the conclusion drawn must at present be in doubt, since there is a very real possibility that misleading selection had occurred in these long-term cultures.

Unfortunately, most of the data so far presented in the literature are quite unsuitable for a formal statistical analysis to decide whether anomalies of the 17,18 chromosomes occur in tumours with a frequency greater than might be due to chance. Since these chromosomes occupy a relatively peripheral position in metaphases prepared by the usual techniques (Miller, Breg *et al.*, 1963), and may occupy a similar position on the mitotic spindle *in vivo*, it might be argued that the

17,18 chromosomes are specially prone to undergo random numerical changes as a result of either mitotic accidents or the methods of preparing cells for study. Such changes would be irrelevant to cancer. However, both the Y chromosome (Miller, Mukherjee *et al.*, 1963), and the heterochromatic X chromosome (Barton, David and Merrington, 1964) occupy similarly peripheral positions and relatively seldom show aberrations in neoplastic cells. A rare exception to this is the occasional loss of the Y chromosome from the tumour cells in chronic granulocytic leukaemia (Speed and Lawler, 1964).

The studies of Kerkis, Radzhabli, Pospelova and Viisotaskoya (1966, personal communication) of the chromosomes involved in the increasing aneuploidy known to occur in human leucocytes with advancing age provide some evidence of the changes that a random process might produce. The loss of chromosomes appeared to be related to their position at metaphase, and the group 17,18 members were quite frequently affected. However, gain of extra group 17,18 chromosomes was not seen and the increase in aneuploidy was in the direction of hypodiploidy. In tumour cells, with the possible exception of the hypotetraploid forms seen in later stages of neoplasia, the gain of extra group 17,18 chromosomes is about as common as loss. Thus it seems most unlikely that either the frequency or the nature of the numerical aberrations of these chromosomes observed in cancer cells is attributable solely to their position on the mitotic spindle.

It has also been argued that many of the chromosome losses and gains seen in cancer cells are completely random results of mitotic accidents, without any special reference to the position of chromosomes on the mitotic spindle. If this were the case, changes should affect the group 6–12 members, 14 in number, more than three times oftener than the group 17,18 chromosomes, of which there are only 4. This is certainly not the case in the large number of malignant lymphomas and related neoplasms which has been studied. In chronic granulocytic leukaemia, numerical changes affecting the chromosomes of the group 17,18 are in fact 6 times more common than changes affecting the chromosomes of group 6–12 (Pedersen, 1969). Finally, the relatively frequent structural rearrangements of group 17,18 chromosomes which have been described (Fig. 1), most of them in several individuals, cannot be explained by mitotic errors. It is exceedingly difficult to attribute these structural anomalies to chance alone, particularly as none of these abnormalities has been observed as an acquired lesion in non-neoplastic tissues. Little doubt can remain that the group 17,18 chromosomes do indeed have some special role in reticuloendothelial neoplasia. The possible nature of this role will next be considered.

*Changes in the group* 17,18 *chromosomes may be of aetiological significance*

Some at least of the changes observed may result from the action of a chemical substance, virus, or other carcinogen and may be the first step in the neoplastic process. It is at present impossible to decide on this point, since in no case do we know the temporal relations between development of the chromosomal anomaly and the appearance of neoplasia. Prospective studies aimed at demonstrating a chromosomal lesion in individuals who subsequently develop lymphoma or other neoplasm are scarcely feasible, because repeated biopsies in normal subjects are unacceptable and there is no known high-risk group which might profitably be studied. Of the anomalies we have described (Fig. 1), the Ep- and Eq- chromosomes might stand the best chance of possessing causal significance, since both are uncommon lesions which cannot be the result of simple mitotic non-disjunction and each may be seen in lymphoma cells without other karyotypic abnormality.

That the Ep- and Eq- anomalies occur in relatively few cases of lymphoma in no way excludes the possibility of their possessing aetiological significance: to apply Koch's postulates to oncogenesis is surely inappropriate. It is hardly to be expected that there should be but a single cytogenetic pathway by which neoplastic status may be reached, even by cells having the same differentiation. A single pathway is even less probable for cells of unlike differentiation. As has been pointed out, most of the anomalies of the group 17,18 chromosomes which occur in tumour cells have also been reported in occasional cases as constitutional chromosomal abnormalities. Although these constitutional aberrations have not been associated with lymphoma or other tumours, this is not a serious argument against a possible role of the acquired form of the anomaly in tumorigenesis. It is possible that the constitutional defect, although on light microscopy morphologically similar to the acquired lesion, is quite different as regards gene constitution. For example, in the constitutional lesion, the apparently deleted chromosomal material may still be present in the cell, translocated onto one of the larger chromosomes and undetectable by ordinary methods of observation. In its new position it may exert genetic effects different from the normal. On the other hand, in the acquired lesion this material may be genuinely lost from the cell. Even if the congenital and the acquired chromosomal anomalies are in fact identical, in the constitutional disorder the anomaly is present in all, or most, of the body cells, and has been present from birth. In the acquired disorders, neither of these considerations applies, and this difference is very profound in itself. Furthermore, the small numbers of individuals possessing the various anomalies in their constitutional form, coupled with their usually short survival due to multiple congenital defects, may have prevented the observation of cancer in any of them.

Thus the possibility cannot be excluded that lesions of the group 17,18 chromosomes, particularly the finer structural alterations such as partial deletions, may have an aetiological role in some tumours.

*Alterations in the group 17,18 chromosomes may be secondary to neoplasia and irrelevant to either the inception or the progression of tumours*

It has been shown that both the frequency and the nature of the abnormalities of group 17,18 chromosomes observed in cancer cells makes them unlikely to be due to chance alone. While accepting the proposition that cytogenetic changes which are both secondary to neoplasia and also irrelevant to its further evolution *may* not depend solely on chance for their occurrence, it seems likely that they will usually be random events. Chromosomal changes which have no bearing on the activity of the tumour in which they arise will tend to be even more diverse than the observed permutations of the 17,18 group, since they will not be liable to the effects of natural selection. However, they are unlikely to be reproduced consistently in many cell generations, and so each anomaly will be found in only a few karyotypes. Such apparently haphazard changes do in fact occur in the karyotypes of some carcinoma cells (Spriggs, Boddington and Clarke, 1962; Ishihara, Kikuchi and Sandberg, 1963). However, alterations of the 17,18 group described in lymphoma (Spiers and Baikie, 1968*a*; Millard, 1968) and leukaemia (Engel, McKee and Bunting, 1967; Spiers and Baikie, 1968*b*) are remarkably consistent, sometimes occurring in every metaphase from a particular tumour. In such circumstances, the changes observed may well be secondary to the onset of neoplasia, but the view that they are irrelevant to the biology of the neoplastic cells is scarcely tenable.

*Anomalies of the group* 17,18 *chromosomes may be secondary phenomena which nevertheless are of evolutionary significance for tumour cells*

While maintaining the view that some of the anomalies observed, particularly the Ep- and Eq- chromosomes, may be primary changes linked to the inception of neoplasia, it seems very probably that some permutations of the 17,18 group are concerned with tumour cell evolution. These changes presumably arise after neoplasia is established, and thus cannot be its cause, but they do influence the subsequent behaviour of the tumour cells in which they occur. This may be the case with various numerical changes, which are seen to affect both normal and structurally anomalous members of the group (Fig. 1). Thus it appears that loss of a group 17,18 chromosome may favour cell proliferation, as cells of this karyotype (C in Fig. 1), are quite often the dominant line in the tumours in which they occur (Spiers and Baikie, 1968*a*, *b*). Structural modifications also may confer a growth

TABLE I.—*Occurrence of Some Structural Anomalies of the Group 17,18 Chromosomes in Neoplastic Cells*

| Chromosomal anomaly | Disease | No. cases with anomaly | Total no. of cases examined* | Authors |
|---|---|---|---|---|
| B. Long-arm isochromosome | Hodgkin's disease | 1 | 1 | Ricci *et al.*, 1962 |
| | Reticulum cell sarcoma | 1 | 2 | Sasaki *et al.*, 1965 |
| | Lymphosarcoma | 1 | 4 | Spiers and Baikie, 1968 |
| | Chronic lymphocytic leukaemia | 1 | 28 | Fitzgerald and Adams, 1965 |
| | Chronic granulocytic leukaemia in metamorphosis | 11 | 24 | de Grouchy *et al.*, 1968 |
| | | 1 | 1 | Engel *et al.*, 1967 |
| | | 1 | 1 | Stich *et al.*, 1966 |
| | Acute granulocytic leukaemia | 1 | 2 | Nowell and Hungerford, 1962 |
| | | 1 | 1 | Engel *et al.*, 1967 |
| D. 3E + Ep- | Hodgkin's disease | 2 | 5 | Spiers and Baikie, 1968 |
| | Follicular lymphoma | 1 | 6 | Spiers and Baikie, 1968 |
| | Reticulum cell sarcoma | 1 | 3 | Millard, 1968 |
| | | 1 | 1 | Kajii *et al.*, 1968 |
| | | ?1 | 4 | Miles *et al.*, 1966 |
| H. 3E + Eq- | Hodgkin's disease | 2 | 8 | Seif and Spriggs, 1967 |
| | Follicular lymphoma | ?1 | 6 | Spiers and Baikie, 1968 |
| | Other lymphoma | 4 | 12 | Millard, 1968 |
| | Acute promyelocytic leukaemia | 1 | 1 | Engel *et al.*, 1967 |
| | Chronic granulocytic leukaemia | 1 | 2 | Lam-Po-Tang, 1967, personal communication |
| | | 1 | 2 | Kiossoglou *et al.*, 1965 |
| M. Translocation to short arms, 3E + Ep+ | Di Guglielmo syndrome | 1 | 1 | Engel *et al.*, 1967 |

* As some communications report single cases, the total number examined is sometimes uncertain.

advantage, and consequent survival advantage, upon tumour cells. The replacement of 1 normal member of the group 17,18 by an Ep- chromosome (D in Fig. 1), or by a long-arm isochromosome (B in Fig. 1), results in the cell becoming monosomic for the short-arm material of the chromosome which has been replaced. In the case of isochromosome formation, the cell also becomes trisomic for the long-arm material (Table II).

Both situations appear favourable for excessive cell multiplication. In chronic granulocytic leukaemia, there is very strong evidence that the formation of an isochromosome for the long arms of a No. 17 chromosome is a secondary phenomenon but relevant to the further evolution of the neoplasm. Thus, in the early stages of the disease, the $Ph^1$ chromosome is the only anomaly present, whilst after the occurrence of metamorphosis to a more anaplastic neoplasm, the isochromosome 17 is observed in a surprisingly high proportion of cases (de Grouchy *et al.*, 1968). When a lymphoma cell apparently loses one No. 17,18 chromosome and acquires 2 Ep- chromosomes (F in Fig. 1), the situation resembles that in cells possessing a long-arm isochromosome, *i.e.* there is a short-arm monosomy and a long-arm trisomy (Table II). In another permutation, (E in Fig. 1), 4 normal group 17,18 chromosomes are retained, and the Ep- is an *extra* chromosome. In this case there is a long-arm trisomy without any short-arm monosomy, a situation which may be particularly advantageous, since cells of this constitution have been observed as the dominant element in a tumour cell population which also contained representatives of cell types (D) and (F) of Fig. 1 (Spiers and Baikie, 1966, 1968*a*).

Paradoxically, gain of a group 17,18 chromosome to produce a fully trisomic condition (G in Fig. 1), seems to be as favourable for cell proliferation as the monosomic or partially monosomic conditions discussed earlier. However, it is possible to formulate an hypothesis which can account for these facts while retaining a reasonable economy of assumptions.

Suppose that the short arms of the affected group 17,18 chromosome carry genes which normally retard cell multiplication, whereas some of the genes on the long arms promote multiplication. Any cytogenetic change which alters the normal 1 : 1 ratio of short-arm to long-arm material in favour of the latter, *e.g.* changes of types (B), (D), (E) or (F) of Fig. 1, will favour cell division, perhaps in an uncontrolled fashion. As may be seen from Table II, in (B), (D), (E) and (F) the short-arm : long-arm ratios for the involved pair, either 17 or 18, are 1 : 3, 1 : 2, 2 : 3 and 1 : 3, respectively. It was earlier pointed out that situation (E), where there is long-arm trisomy *without* short-arm monosomy, may be particularly advantageous for cell growth and survival, as cells of this constitution appear to

TABLE II.—*Relative Amounts of Long-arm and Short-arm Material of the Affected Chromosome Pair in Some of the Group* 17,18 *Anomalies Depicted in Fig.* 1

| Anomaly | Short-arm material (SA) | Long-arm material (LA) | SA : LA Ratio |
|---|---|---|---|
| A | Disomic | Disomic | 1 : 1 |
| B | Monosomic | Trisomic | 1 : 3 |
| C | Monosomic | Monosomic | 1 : 1 |
| D | Monosomic | Disomic | 1 : 2 |
| E | Disomic | Trisomic | 2 : 3 |
| F | Monosomic | Trisomic | 1 : 3 |
| G | Trisomic | Trisomic | 1 : 1 |

compete successfully with cells which do show a short-arm monosomy. This is readily accounted for by the very reasonable assumption that some of the genetic material on the short arms is of importance in cell metabolism and operates more effectively when in the disomic state. Such type (E) cells will possess an excess of " proliferative " genes due to their long-arm trisomy while having a more efficient metabolism than, say, type (D) cells because no important genetic material is missing from the cell.

The above hypothesis does not, of course, by itself explain why the chromosomal constitutions (C) and (G) of Fig. 1 might favour cell multiplication, since in each situation the short-arm : long-arm ratio is 1 : 1 as in normal cells (Table II). Since gene-dose effects do not always follow the principles of simple arithmetic, it could be postulated that the " anti-proliferative " genes on the short arms are virtually ineffective when monosomic, whilst their effect is not augmented above the normal by becoming trisomic. If the " proliferative " genes on the long arms were subject to no such limitation, the *effective* short-arm : long-arm ratios in (C) and (G) type cells would be 0 : 1 and 2 : 3 respectively. Because of insufficient evidence, this view is merely a speculation, and in fact, a simpler explanation is available. On reviewing those cases of malignant lymphoma (Sandberg *et al.*, 1964; Miles, Geller and O'Neill, 1966; Sinks and Clein, 1966; Miles, 1967; Millard, 1968; Spiers and Baikie, 1968*a*) and leukaemia (Court Brown and Tough, 1963; Pedersen, 1964; de Grouchy *et al.*, 1966; Spiers and Baikie, 1968*b*) in which cells of types (C) and (G) have been observed, it was found that changes in other chromosomal groups were usually present. These changes were often multiple and complex and sometimes included the possession of 1 or more $Ph^1$ chromosomes. By contrast, the Ep- chromosomal anomaly commonly occurs in cells whose chromosomal constitution is otherwise normal or nearly so. Thus the effects of trisomy or monosomy of a group 17,18 chromosome may be less important, and possibly mediated in a quite different way, to the effects of structural anomalies of these chromosomes, since the 2 types of aberration seem to occur in cells whose other genetic components are very different.

The foregoing arguments cannot apply to the Eq- chromosome described by Millard (1968) and by Seif and Spriggs (1967), since here the deletion involves the *long* arms of a group 17,18 chromosome, probably No. 18. The Eq- chromosome has been observed as an extra element (I) and also in place of one of the normal chromosomes of the group (H in Fig. 1). If this change is of evolutionary significance for the neoplastic cells, which seems probable, it may be necessary to postulate other inhibitory or regulatory genes, located on the distal part of the long arm of No. 18, whose loss favours cell growth. A similar situation may well obtain in chromosome 21, where formation of the $Ph^1$, by deletion of part of the long arms, clearly favours cell proliferation.

The objection may be raised that all the above suggestions are based on relatively slender evidence and that they invest the group 17,18 chromosomes with complex structural and functional attributes. This is quite true. On the other hand, there is sufficient evidence which seems to implicate these chromosomes in the genesis and/or evolution of some malignant neoplasms to warrant a tentative hypothesis to explain the facts. Such an hypothesis might serve as a basis for further and better studies leading to its modification or rejection. The complexity of the hypothesis is unfortunate but accords well with the habitual complexity of biological systems in general and of cancer in particular.

### REFERENCES

BAIKIE, A. G.—(1966) *Acta haemat.*, **36**, 157.

BAIKIE, A. G., COURT BROWN, W. M., BUCKTON, K. E., HARNDEN, D. G., JACOBS, P. A. AND TOUGH, I. M.—(1960) *Nature Lond.*, **188**, 1165.

BAIKIE, A. G. AND SPIERS, A. S. D.—(1966) *Lancet*, i, 985.

BARTON, D. E., DAVID, F. N. AND MERRINGTON, M.—(1964) *Ann. hum. Genet.*, **28**, 123.

BERGER, R.—(1965) *Annls Génét.*, **8**, 70.

BÜHLER, E. M., BÜHLER, U. K. AND STADLER, G. R.—(1964) *Lancet*, i, 170.

CHICAGO CONFERENCE—(1966) Birth Defects: original article series, *II*. 2. New York (The National Foundation).

COURT BROWN, W. M. AND TOUGH, I. M.—(1963) *Adv. Cancer Res.*, **7**, 351.

CRAWFORD, M. D'A.—(1961) *Lancet*, ii, 22.

ENGEL, E., MCGEE, B. J., HARTMANN, R. C. AND MONTMOLLIN, M. E.—(1965) *Cytogenetics*, **4**, 157.

ENGEL, E., MCKEE, L. C. AND BUNTING, K. W.—(1967) *Lancet*, ii, 42.

FEINGOLD, M. AND SCHWARTZ, R. S.—(1968) *Lancet*, ii, 1086.

FEINGOLD, M., SCHWARTZ, R. S., ATKINS, L., ANDERSON, R., BARTSOCAS, C. S., PAGE, D. L. AND LITTLEFIELD, J. W.—(1968) *J. clin. Invest.*, **47**, 34.

FINLEY, S. C., FINLEY, W. H., NOTO, T. A., UCHIDA, I. A. AND RODDAM, R. F.—(1968) *Lancet*, i, 1095.

FITZGERALD, P. H. AND ADAMS, A.—(1965) *J. natn. Cancer Inst.*, **34**, 827.

GENEST, P., LECLERC, R. AND AUGER, C.—(1963) *Lancet*, i, 1426.

GOFAM, J. W., MINKLER, J. L. AND TANDY, R. K.—(1967) UCRL-50356, Clearing-house for Federal Scientific and Technical Information, National Bureau of Standards, Springfield, Virginia, U.S.A.

DE GROUCHY, J.—(1965). *J. Pediat.*, **66**, 414.

DE GROUCHY, J., LAMY, M., THIEFFRY, S., ARTHUIS, M. AND SALMON, C.—(1963) *C.r. hebd. Séanc. Acad. Sci., Paris*, **256**, 1028.

DE GROUCHY, J., DE NAVA, C., CANTU, J. M., BILSKI-PASQUIER, G. AND BOUSSER, J.—(1966) *Am. J. hum. Genet.*, **18**, 485.

DE GROUCHY, J., DE NAVA, C., FEINGOLD, J., BILSKI-PASQUIER, G. AND BOUSSER, J.—(1968) *Eur. J. Cancer*, **4**, 481.

HECHT, F., BRYANT, J. S., MOTULSKY, A. G. AND GIBLETT, E. R.—(1963) *J. Pediat.*, **63**, 605.

HUMAN CHROMOSOMES STUDY GROUP—(1960) *Cerebr. Palsy Bull.*, **2**, No. 3, Supplement.

ILBERY, P. L. T. AND ALEXANDER, J. M.—(1967) *Australas. Ann. Med.*, **16**, 215.

ISHIHARA, T., KIKUCHI, Y. AND SANDBERG, A. A.—(1963) *J. natn. Cancer Inst.*, **30**, 1303.

JACOBSEN, P.—(1966) *Lancet*, i, 1379.

KADOWAKI, J.-I., THOMPSON, R., ZUELZER, W. W., WOOLEY, P. V., BROUGH, A. J. AND GRUBER, D.—(1965) *Lancet*, ii, 1152.

KAJII, T., NEU, R. L. AND GARDNER, L. I.—(1968) *Cancer, N.Y.*, **22**, 218.

KIOSSOGLOU, K. A., MITUS, W. J. AND DAMESHEK, W.—(1965) *Lancet*, ii, 665.

LAWLER, S. D., PENTYCROSS, C. R. AND REEVES, B. R.—(1968) *Br. med. J.*, ii, 213.

LUBS, H. A., SALMON, J. H. AND FLANIGAN, S.—(1966) *Cancer, N.Y.*, **19**, 591.

MILES, C. P.—(1967) *Cancer, N.Y.*, **20**, 1253.

MILES, C. P., GELLER, W. AND O'NEILL, F.—(1966) *Cancer, N.Y.*, **19**, 1103.

MILLARD, R. E.—(1968) *Eur. J. Cancer*, **4**, 97.

MILLARD, R. E. AND SEIF, G.—(1967). *Lancet*, i, 781.

MILLER, O. J., BREG, W. R., MUKHERJEE, B. B., GAMBLE, A. VAN N. AND CHRISTAKOS, A. C.—(1963) *Cytogenetics*, **2**, 152.

MILLER, O. J., MUKHERJEE, B. B., BREG, W. R. AND GAMBLE, A. VAN N.—(1963) *Cytogenetics*, **2**, 1.

MOOREHEAD, P. S. AND SAKSELA, E.—(1963). *J. cell. comp. Physiol.*, **62**, 57.

NOWELL, P. C. AND HUNGERFORD, D. A.—(1960*a*). *J. natn. Cancer Inst.*, **25**, 85.—(1960*b*) *Science, N.Y.*, **132**, 1497.—(1962) *J. natn. Cancer Inst.*, **29**, 911.—(1964) *Ann. N.Y. Acad. Sci.*, **113**, 654.
PATAU, K.—(1960) *Am. J. hum. Genet.*, **12**, 250.
PEDERSEN, B.—(1964) *Acta path. microbiol. scand.*, **61**, 497.—(1967) *Acta path. microbiol. scand.*, **69**, 192.—(1969) 'Cytogenetic Evolution in Chronic Myelogenous Leukaemia'. Copenhagen (Munksgaard).
RICCI, N., PUNTURIERI, E., BOSI, L. AND CASTOLDI, G. L.—(1962) *Lancet*, ii, 564.
RICHARDS, B. W. AND HOBBS, J. R.—(1968) *Lancet*, i, 1426.
SANDBERG, A. A., ISHIHARA, T., CROSSWHITE, L. H. AND HAUSCHKA, T. S.—(1962) *Blood*, **20**, 393.
SANDBERG, A. A., ISHIHARA, T., KIKUCHI, Y. AND CROSSWHITE, L. H.—(1964) *Cancer, N.Y.*, **17**, 738.
SASAKI, M. S., SOFUNI, T. AND MAKINO, S.—(1965) *Cancer, N.Y.*, **18**, 1007.
SEIF, G. S. F. AND SPRIGGS, A. I.—(1967) *J. natn. Cancer Inst.*, **39**, 557.
SINKS, L. F. AND CLEIN, G. P.—(1966) *Br. J. Haemat.*, **12**, 447.
SPEED, D. E. AND LAWLER, S. D.—(1964) *Lancet*, i, 403.
SPIERS, A. S. D. AND BAIKIE, A. G.—(1966) *Lancet*, i, 506.—(1967) *Br. J. Cancer*, 21, 679. (1968*a*) *Cancer, N.Y.*, **22**, 193.—(1968*b*) *Br. J. Cancer*, **22**, 192.
SPRIGGS, A. I., BODDINGTON, M. M. AND CLARKE, C. M.—(1962) *Br. med. J.*, ii, 1431.
STEWART, J., GO, S., ELLIS, E. AND ROBINSON, A.—(1968) *Lancet*, ii, 779.
STICH, H. F., BACK, F., DORMER, P. AND TSIRIMBAS, A.—(1966) *Klin. Wschr.*, **44**, 334.
SUMMIT, R. L.—(1964). *Cytogenetics*, **3**, 201.
TANZER, J., BOIRON, M., LEVY, D., SELIGMANN, M. AND BERNARD, J.—(1966) *XIth Int. Cong. Haemat.* 319, Sydney (The Government Printer).
TJIO, J. H., MARSH, J. C., WHANG, J. AND FREI, E.—(1963) *Blood*, **22**, 178.
UCHIDA, I. A., MCRAE, K. N., WANG, H. C. AND RAY, M.—(1965) *Am. J. hum. Genet.*, **17**, 410.
VAN DYKE, H. E., VALDMANIS, A. AND MANN, J. D.—(1964) *Am. J. hum. Genet.*, **16**, 364.
WANG, H. C., MELNYK, J., MCDONALD, L. T., UCHIDA, I. A., CARR, D. H. AND GOLDBERG, B.—(1962) *Nature, Lond.*, **195**, 733.
WARREN, R. J.—(1968) *Lancet*, ii, 350.